# A phase 2a randomised, double-blind, placebo-controlled, parallel-group, add-on clinical trial of ebselen (SPI-1005) as a novel treatment for mania or hypomania

**DOI:** 10.1007/s00213-020-05654-1

**Published:** 2020-09-09

**Authors:** Ann L Sharpley, Clare Williams, Adele A Holder, Beata R Godlewska, Nisha Singh, Milensu Shanyinde, Orla MacDonald, Philip J Cowen

**Affiliations:** 1 Neurosciences Building, Department of Psychiatry, University of Oxford, Warneford Hospital, Oxford, OX3 7JX UK; 2 Oxford Health NHS Foundation Trust, Warneford Hospital, Oxford, UK; 3 Nuffield Department of Primary Care Health Sciences, University of Oxford, Oxford, UK

**Keywords:** Ebselen, Randomised clinical trial, Bipolar, Mania, Add-on, YMRS, Lithium mimetic

## Abstract

**Rationale:**

Lithium is an effective prophylactic and anti-manic treatment in bipolar disorder; however, its use is declining through perceived poor tolerance and toxicity. Lithium inhibits inositol monophosphatase (IMPase), a probable key therapeutic mechanism. The anti-inflammatory drug, ebselen, also inhibits IMPase and appears well-tolerated and safe.

**Objectives:**

To assess the efficacy of adjunctive ebselen in mania using the Young Mania Rating Scale (YMRS) (primary outcome) and the Altman Self-Rating Mania (ASRM) Scale and Clinical Global Impression-Severity Scale (CGI-S) among the secondary outcomes.

**Methods:**

Randomised, double-blind, placebo-controlled, parallel-group trial conducted between October 2017 and June 2019, at Oxford Health NHS Foundation Trust. Pharmacy-controlled randomisation was computer-generated, with full allocation concealment. In/outpatients (*n* = 68) aged 18–70, experiencing mania or hypomania, were assigned to 3 weeks ebselen (600 mg bd) (*n* = 33) or placebo (*n* = 35). Participants received usual clinical care and psychotropic medication.

**Results:**

Ebselen was numerically, but not statistically, superior to placebo in lowering scores on the YMRS (adjusted mean difference and 95% confidence interval, − 1.71 (− 5.34 to 1.91), *p* = 0.35) and ASRM (− 1.36 (− 3.75 to 1.17), *p* = 0.29). However, scores on the CGI-S were significantly lower at week 3 in ebselen-treated participants (adjusted mean difference, − 0.58 (− 1.14 to − 0.03), *p* = 0.04). A post hoc analysis excluding patients taking concomitant valproate treatment magnified the difference between ebselen and placebo on the YMRS. Adverse events were comparable between groups, and mild.

**Conclusions:**

Ebselen merits further investigation where concomitant psychotropic medication is better controlled and participants taking valproate are excluded. If effective, ebselen’s superior tolerance and safety could make it a useful alternative to lithium.

**Trial registration:**

Trial Registry: www.clinicaltrials.gov, Identifier: NCT03013400.

**Electronic supplementary material:**

The online version of this article (10.1007/s00213-020-05654-1) contains supplementary material, which is available to authorized users.

## Introduction

Lithium is the most effective and widely recommended prophylactic drug treatment for patients with bipolar disorder (Cousins et al. 2020). Lithium is also effective in the treatment of acute mania both as a monotherapy and as adjunctive treatment (Geddes and Miklowitz 2013). However, lithium is not well tolerated, requires regular medical monitoring including blood tests and has a narrow therapeutic index. Furthermore, long-term lithium treatment is associated with renal impairment in a significant minority of patients (Harrison et al. 2018). Not surprisingly, many patients are reluctant to take lithium despite its benefits. For all these reasons, the use of lithium is in decline (Zivanovic 2017).

There would therefore be great advantages in identifying a drug that had the same action and clinical benefit as lithium but was better tolerated and safer. Lithium has several putative pharmacological targets, but in vitro and animal experimental studies converge to suggest that inhibition of inositol monophosphatase (IMPase) may be critical to its therapeutic action. By inhibiting this enzyme, lithium decreases inositol recycling, thereby diminishing second messenger signalling at overactive synapses (Berridge et al. 1989; Atack 1996; Agam et al. 2009). Nevertheless, it is important to note that other molecular targets have been convincingly implicated in lithium’s mode of action, for example, inhibition of glycogen synthase kinase-3 (GSK3) (for a recent review, see Haggarty et al. 2020).

Singh et al. (2013) probed the National Institutes of Health Clinical Collection, a library of bioavailable drugs considered clinically safe, and identified ebselen, as a potent IMPase inhibitor. Singh et al. (2013) showed that ebselen decreased inositol recycling in mice and reproduced several effects of lithium in animal models, for example, diminishing the functional sensitivity of 5-HT_2A_ receptors and inhibiting the hyperactivity that follows amphetamine administration—a putative model of mania. These effects of ebselen were reversed by cerebral inositol administration indicating mediation by disruption of inositol recycling in the brain. Ebselen is a selenium-containing small molecule, with broad anti-inflammatory activity. Ebselen has potent glutathione peroxidase (GPx) like activity and was shown to induce GPx1 in the inner ear under redox stress (Kil et al. 2007). Ebselen has been studied in several phase 3 trials in Japan as a neuroprotective treatment to limit the neurological deficits produced by acute stroke and in several phase 2 trials in the USA as a treatment for hearing loss and tinnitus disorders (Kil et al. 2017).

Subsequently our group found in two placebo-controlled studies involving a total of 60 healthy participants that ebselen (600 mg twice daily for 2 days and 1200 mg twice daily for 2 days) lowered the concentration of inositol in anterior cingulate cortex measured by magnetic resonance spectroscopy (MRS) (Singh et al. 2016; Masaki et al. 2016a). This finding indicates that a dose of ebselen of 1200 mg daily is sufficient to interact with the proposed IMPase target in the human brain and thereby decrease the availability of inositol. In these studies, ebselen produced other central effects relevant to mood disorder, for example, increasing learning in a reward/punishment task and modifying the recognition of emotional facial expressions (Singh et al. 2016; Masaki et al. 2016b).

Developing a mood stabiliser for bipolar disorder is challenging and the pharmaceutical industry has largely withdrawn from this field. Because of the need for sustained follow-up, long-term studies of a potential prophylactic agent are unlikely to be conducted without initial evidence of potential therapeutic efficacy. Lithium is an effective acute anti-manic agent, but placebo-controlled trials of mania are difficult to carry out in standard clinical settings. Therefore, we conducted a short-term, placebo-controlled, ‘add-on’ study of ebselen in patients with hypomania/mania receiving standard drug treatment for a manic episode. This approach has been used successfully to detect the anti-manic effect of mood stabiliser treatments including lithium (Chou et al. 1999) and carbamazepine (Klein et al. 1984).

## Methods

### Trial design

This study was a phase 2a, single centre, 3-week, double-blind, parallel-group, placebo-controlled, add-on randomised clinical trial (RCT) testing ebselen or placebo. Participants were recruited from October 2017 to June 2019 at Oxford Health NHS Foundation Trust, UK. The trial was funded by the Stanley Medical Research Institute (SMRI) (14T-005) and supported by the National Institute of Health Research (NIHR) Oxford Health Biomedical Research Centre (OHBRC). Our collaborators, Sound Pharmaceuticals Inc. (SPI), Seattle, USA, supplied ebselen (SPI-1005) and matching placebo and cross referenced four active indications (INDs) in the USA where SPI-1005 had been tested for 3–4 weeks in several hearing loss and tinnitus indications. The trial had ethics approval (National Research Ethics Service Committee South Central – Oxford C: 16/SC/0553) and is registered with the US National Institutes of Health (NIH) (www.clinicaltrials.gov Identifier: NCT03013400). Ebselen or placebo was added to any ongoing pharmacological treatment that patients were receiving from their clinical team. The protocol and statistical analysis plan (SAP) are available in the Supplementary Material online.

### Inclusion/exclusion criteria

Participants with bipolar disorder, who were currently experiencing mania/hypomania, were included. Specifically, eligible participants were aged between 18 and 70 years old; fulfilled The Diagnostic and Statistical Manual of Mental Disorders (5th ed.; DSM-5; American Psychiatric Association 2013) criteria for a Manic or Hypomanic Episode; used effective contraception; allowed contact with the clinical care team to ensure that they were in agreement for the participant to be included in the trial; and had capacity to provide written informed consent and to follow trial procedures.

Participants were excluded if they were taking lithium; female and were pregnant, lactating or planning pregnancy; had known significant renal or hepatic impairment; had scheduled elective surgery or procedures requiring general anaesthesia; had any other significant disease or disorder; had participated in another trial involving an investigational product in the previous 12 weeks; had clinically significant illicit substance or alcohol misuse; and had previously been randomised to this trial.

### Outcome measures

At screening, participants fulfilled DSM-5 criteria for a Manic or Hypomanic Episode. The primary outcome was change in the clinician-rated Young Mania Rating Scale (YMRS) (range 0 to 60) from baseline to follow-up. Higher scores indicate more severe symptoms of mania (Young et al. 1978). The secondary outcome measures were as follows: questionnaires: Altman Self-Rating Mania (ASRM) (Altman et al. 1997); Clinical Global Impression (CGI) - Severity (CGI-S) and Improvement (CGI-I) (Guy 1976); Hamilton Depression Rating Scale (HDRS 17) (Hamilton 1960); Quick Inventory of Depressive Symptomology-Self-Report (QIDS-SR-16) (Rush et al. 2003) and Leeds Sleep Evaluation Questionnaire (LSEQ) (Parrott and Hindmarsh 1980). Allocation concealment was assessed at study endpoint asking participant, researcher and, if appropriate, relative/key carer to guess whether they had been assigned ‘active’ or ‘placebo’ and the certainty of this allocation. Concomitant medication and adverse events (AEs) were noted throughout the trial. Compliance was measured by capsule count. An actigraph monitor (Motion Watch 8; CamNtech Ltd. Cambridgeshire, UK) worn continuously allowed changes in activity to be monitored. An optional blood sample was collected to assess ebselen levels in plasma to confirm exposure. The results of these assays are currently not available due to analysis issues and will be reported elsewhere.

### Randomisation, allocation concealment and study interventions

The pharmacy-controlled computer-generated randomisation algorithm minimised separately on two variables, gender and YMRS score (≥ 20 and < 20). Eligible participants were randomised in a 1:1 allocation with subject numbers assigned sequentially as each participant entered the trial. There was no run-in period. No code break occurred during this trial. Quadruple masking took place with participants, research team, clinical team and analysis all blind to treatment allocation. Participants were required to take three capsules twice per day (morning and evening) with a daily dose of 1200 mg/day of ebselen (SPI-1005). Placebos containing the same three excipients in the active capsules were matched in colour, size and shape.

### Procedure

Participants were identified from the clinical caseloads of the general adult and old age psychiatric teams by NHS research facilitators embedded within the clinical teams, as well as clinicians and care co-ordinators. Potential participants were given a participant information sheet (PIS) and, with their permission, the research team were notified. Interested individuals were also able to contact a member of the research team directly from posters placed in clinical settings, online clinical trial registration or recruitment websites (for example, University Department of Psychiatry and NHS research websites) or through patient and public involvement events (PPI). Once contacted, a research team member liaised directly with the participant to discuss the study and, if they fulfilled general criteria for inclusion, and continued to express an interest, the researcher arranged a screening visit. Visits took place at inpatient or outpatient facilities, at the research team base or in an individuals’ home. Potential participants were given adequate time to reflect on the information, had any questions answered and had capacity to give written informed consent. In addition to the screening questionnaires, other health domain data collected included demographic data and general health and medication history.

If eligible, participants were randomly allocated to a treatment group and commenced treatment that day or the following morning. Following the screening visit (visit 1), there were a total of four further visits, at weekly intervals, where the same clinician- and self-rated questionnaires were repeated. The final ratings were taken at week 4 (visit 5), 1 week after cessation of ebselen/placebo treatment. Data collected were entered in paper case record forms (CRFs) with questionnaires completed on iPads using Qualtrics (a subscription software platform) or on paper depending on the participants’ choice.

Compliance, changes in concomitant medication and adverse events were elicited at each visit. At follow-up (visit 5), participants were, in addition, asked to complete an allocation guess questionnaire and provide study feedback. Participants received £20 per visit in addition to travel expenses.

### Statistical analysis

Analysis was conducted blinded to treatment allocation following the analysis pre-specified in the statistical analysis plan (Supplementary Material online) using STATA version 16.0. Research team members uploaded data, reconciled data queries and conducted quality assurance checks on the database. All data cleaning checks were performed before the final data lock.

Analysis of the primary outcome (YMRS) was undertaken using a mixed effects linear model fitted to the data. This model utilised all data collected at all time points following randomisation. Fixed effects included randomised group, severity of manic symptoms on YMRS at baseline, psychosis, gender, time and time by randomised group interaction. This allowed assessment of treatment effect at each time point. An unstructured variance covariance matrix was specified between repeated measures on the same individual. A random effect accounted for repeated measures from the same participant. The adjusted mean difference in YMRS scores between randomised groups was reported for each time point alongside a 95% confidence interval and *p* value. Secondary outcomes of mood questionnaires were analysed in the same way. The primary outcome analysis was repeated in a post-unblinding secondary analysis to determine whether YMRS improvements between groups were altered if participants receiving concomitant treatment with the mood stabiliser and anti-manic agent valproate were excluded. Like lithium, the therapeutic mechanism of valproate may involve changes in inositol cycling (Rosenberg 2007).

An a priori power calculation was undertaken. The YMRS is frequently used as an endpoint in studies of pharmacologic treatments of mania and a difference of six points on the YMRS at endpoint indicates a clinically significant effect of an active treatment (Lukasiewicz et al. 2013). A power of 0.8 at a *p* < 0.05 level of significance is achieved with 25 participants per group. A sample size of 60 allowed for a 15% dropout rate, which is reasonable for a 3-week period of study treatment.

## Results

### Participant characteristics

**Fig. 1:**
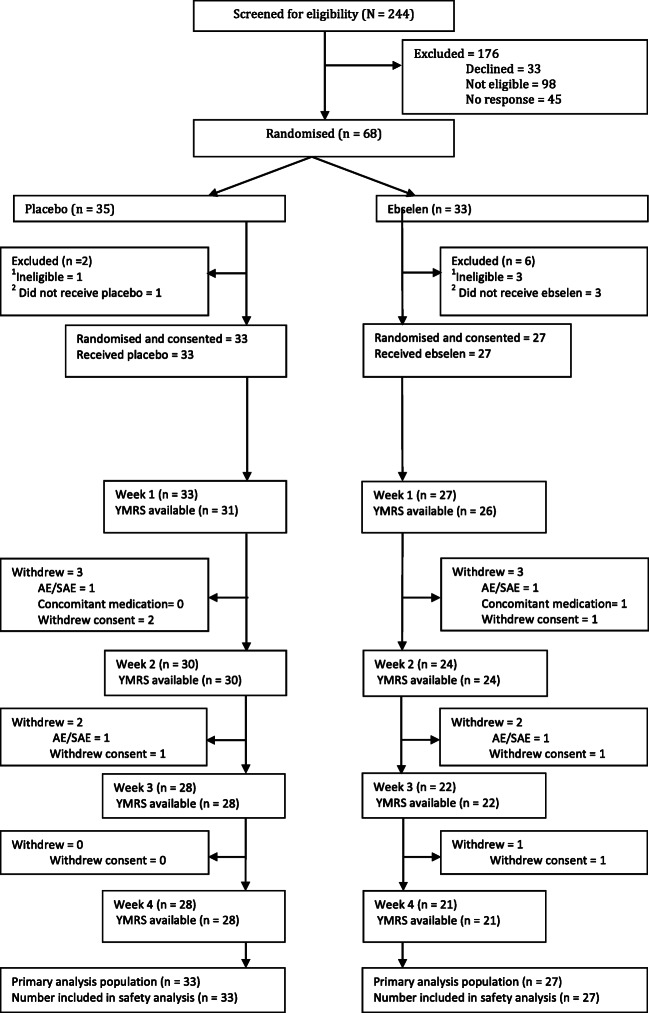
Consort flow diagram: study sample and patient throughput. ^1^In four participants, medication was ordered due to distance. However, during screening visit, *n* = 2, found to be ineligible; *n* = 1, unable to give informed consent and would not comply with trial procedures and *n* = 1 decided to not participate in the trial. ^2^As per definition of inclusion in analysis, 4 people did not start taking their medication (*n* = 3 withdrew consent before they started and *n* = 1, care team was not in agreement with patient participating in the trial)

After screening visit exclusions, 68 participants were randomised to either ebselen (*n* = 33) or placebo (*n* = 35) (see CONSORT; Fig. 1). All analyses are based on the 60 participants (ebselen *n* = 27 or placebo *n* = 33) who were randomised and started treatment (intent-to treat). Fifty participants were included in the end of treatment week 3 YMRS analysis (ebselen *n* = 22; placebo *n* = 28) and overall, 82% of randomised participants completed the study (ebselen *n* = 21, *n* = 28 placebo).

Demographic and clinical characteristics of each group are shown in Table 1. The majority were inpatients, probably accounting for the relatively high frequency of psychotic symptoms (around 40%). It was often only possible to approach inpatients after they had started to improve clinically, so participation in the trial occurred against a background of developing symptom resolution. The majority in both treatment groups were female patients, who when approached, tended to be willing to consider participation, while the male patients had higher levels of irritability and were less inclined to engage. Most participants (91% placebo, 89% ebselen) were already receiving treatment with antipsychotic drugs, with a smaller proportion taking mood stabilisers, either lamotrigine or valproate (24% placebo, 41% ebselen) (Table 1). Overall, mean compliance (judged by capsule count) was 80% in the placebo group and 71% in the ebselen group.

**Table 1:** Demographic and clinical features of study participants

	Placebo (*n* = 33)	Ebselen (*n* = 27)
Age in years		
Mean (SD)	41.2 (14.4)	43.7 (14.2)
Range	[20 to 68]	[20 to 69]
Gender, *n* (%)		
Female	23 (70)	21 (85)
Male	10 (30)	6 (15)
Baseline YMRS score		
Mean (SD)	18 (5.9)	20 (6.1)
Range	9 to 29	8 to 29
BMI		
Mean (SD)	27.3 (7.0)	27.2 (5.7)
Range	19.1 to 56.6	18.9 to 46.3
Smoker, *n* (%)		
Yes	17 (52)	13 (48)
No	16 (48)	14 (52)
In/out patient status, *n* (%)		
In	19 (58)	21 (78)
Out	14 (42)	6 (22)
Psychosis symptoms, *n* (%)		
Yes	14 (42)	11 (41)
No	19 (58)	16 (59)
Concomitant medication *n* (%)		
Antipsychotic^1^	30 (91)	24(89)
Mood stabiliser^2^	8 (24)	11(41)
Benzodiazepine	19 (58)	15 (56)

*BMI* body mass index; *YMRS* Young Mania Rating Scale

^1^Placebo patients: olanzapine (12), aripiprazole (7), quetiapine (6), risperidone (5), haloperidol (1), amisulpride (1), paliperidone (1), zuclopenthixol (1). Ebselen patients: olanzapine (9), aripiprazole (10), quetiapine (5), risperidone (3), paliperidone (3). Some patients were taking more than one antipsychotic drug

^2^Placebo patients: valproate (6), lamotrigine (2). Ebselen patients: valproate (5), lamotrigine (6)

### Primary outcome

**Fig. 2:**
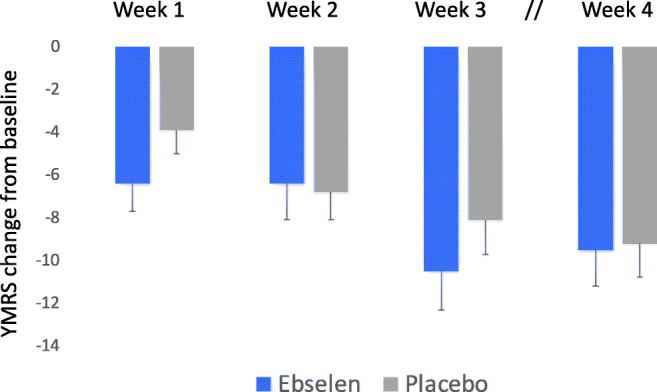
Non-adjusted mean (SEM) change from baseline scores on the Young Mania Rating Scale (YMRS) following addition of ebselen (600 mg bd) (*n* = 27) or placebo (*n* = 33) to the treatment of patients with mania/hypomania. There were no statistically significant differences at any of the time points

**Fig. 3:**
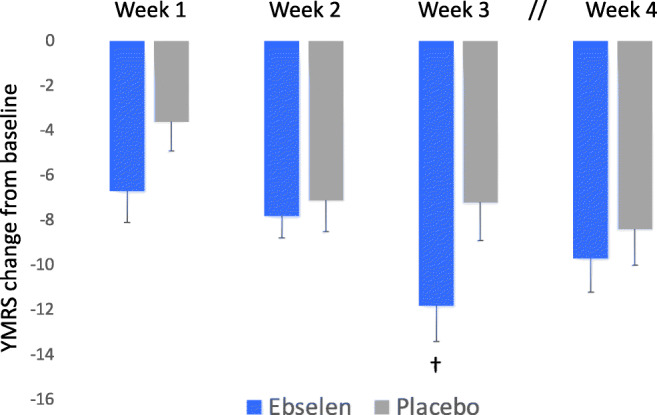
Non-adjusted mean (SEM) change from baseline scores on the Young Mania Rating Scale (YMRS) following addition of ebselen (600 mg bd) (*n* = 22) or placebo (*n* = 27) to the treatment of patients with mania/hypomania, excluding patients taking valproate. “†” The adjusted mean difference and 95% confidence interval (CI) (change from baseline) in YMRS score between the two groups at 3 weeks was − 3.67 (− 7.39 to 0.06) (*p* = 0.054)

Both treatment groups improved symptomatically during the study. Those taking ebselen had lower YMRS scores after 3 weeks of treatment, although this was not statistically significant when compared with placebo. The adjusted mean difference and 95% confidence interval (CI) (change from baseline) in YMRS score between the two groups at 3 weeks was − 1.71 (− 5.34 to 1.91), *p* = 0.35 (Fig. 2). Eleven of the participants (*n* = 6 placebo, *n* = 5 ebselen) were taking valproate. Since the latter drug, like lithium, is considered a mood stabiliser and anti-manic agent, and may act to inhibit inositol metabolism (Rosenberg 2007), a post hoc unblinded analysis was conducted where the primary analysis was repeated with the valproate-treated participants excluded. An improvement in YMRS scores in the ebselen group, of borderline significance, was seen at 3 weeks (adjusted means and 95% CI, − 3.67 (− 7.39 to 0.06), *p* = 0.054) (Fig. 3). Interestingly at week 4, after 1 week of withdrawal of adjunctive treatment, numerically, the placebo group continued to improve on the YMRS while the ebselen group did not (Figs. 2 and 3), and showed some loss of clinical benefit at week 4.

### Secondary outcomes

**Fig. 4:**
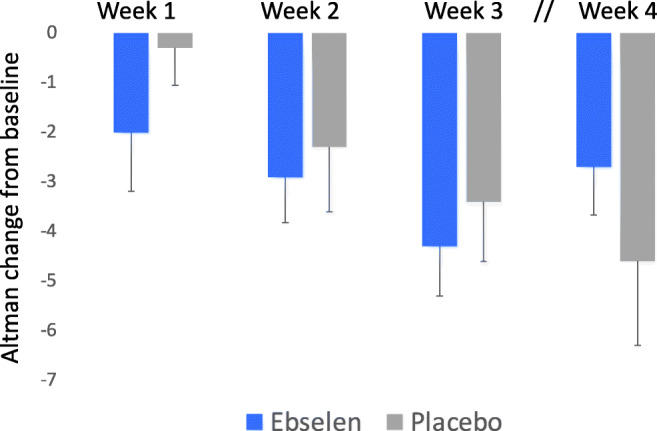
Non-adjusted mean (SEM) change from baseline scores on the Altman Self Rating Scale (ASRM) following the addition of ebselen (600 mg bd) (*n* = 27) or placebo (*n* = 33) to the treatment of patients with mania/hypomania. There were no statistically significant differences at any time points

**Fig. 5:**
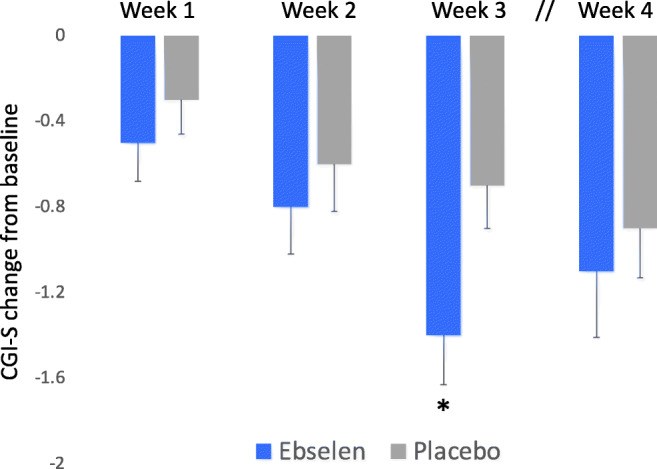
Non-adjusted mean (SEM) change from baseline scores on the Clinical Global Impression Severity (CGI-S) scale following the addition of ebselen (600 mg bd) (*n* = 27) or placebo (*n* = 33) to the treatment of patients with mania/hypomania. “*” The adjusted mean difference and 95% confidence interval (CI) (change from baseline) in CGI-S score between the two groups at 3 weeks was − 0.58 (− 1.14 to − 0.03); *p* = 0.040

A similar pattern of response to that on the YMRS was seen on the self-rated ASRM (Fig. 4). At 3 weeks, ebselen-treated participants showed a numerically greater fall in score on the ASRM but this was not statistically significant (adjusted means and 95% CI, − 1.36 (− 3.75 to 1.17), *p* = 0.29). However, on the CGI-S, the reduction in score of the ebselen group at week 3 was significantly greater than the placebo group (adjusted mean difference, − 0.58 (− 1.14 to − 0.03), *p* = 0.04) (Fig. 5). In both these measures, the same pattern of change was seen at week 4, with the placebo group continuing to improve numerically while those taking ebselen showed some lessening of clinical response. Other secondary outcomes are shown in the Supplementary Material online and actigraphy data will be published elsewhere. However, we found no significant difference between ebselen and placebo-treated participants on the HDRS-17 or the QIDS-SR-16 (Supplementary Figures 1 and 2). There were also few significant differences on the LSEQ with the quality of sleep (QOS) measure being significantly worse in ebselen-treated participants relative to placebo during the first week of treatment but significantly better during week 3 (Supplementary Table 2).

There was one serious adverse event (SAE) in the placebo group where a participant required hospitalisation. There was no statistically significant difference between the groups in terms of participants who reported AEs (Chi square test; *p* = 0.489) (Table 2). Table 3 shows common AEs, by randomised group, with headache reported as the most common AE in approximately 30% of participants. There were no significant differences between ebselen and placebo, and no particular profile of AEs associated with ebselen.

**Table 2:** Adverse events (AEs) reported by participants

	Placebo *n* = 33 (%)	Ebselen *n* = 27 (%)
Randomised participants with		
At least one AE	29 (87.9)	22 (81.5)
No AEs	4 (12.1)	5 (18.5)
Chi square test		0.489
Number of AEs per participant		
0	4 (12.1)	5 (18.5)
1	6 (18.2)	4 (14.8)
2	5 (15.2)	3 (11.1)
3	4 (12.1)	4 (14.8)
> 3	14 (42.4)	11 (40.7)
Total number of AEs	124	94

**Table 3:** Summary of common adverse events (AEs) by randomised group

Common AEs	*n* events	Placebo *n* = 33 (%)	*n* events	Ebselen *n* = 27 (%)
Headache	16	12 (36)	11	8 (30)
Drowsiness	5	4 (12)	9	7 (26)
Dizziness	7	4 (12)	4	3 (11)
Anorexia	5	5 (15)	4	3 (11)
Nausea	6	5 (15)	3	3 (11)
Pruritus	4	3 (9)	4	4 (15)
URTI	2	2 (6)	6	5 (19)
Abdominal pain	4	3 (9)	3	3 (11)
Vomiting	4	3 (9)	2	2 (7)
Back pain	3	3 (9)	2	2 (7)
Diarrhoea	5	4 (12)	0	0 (0)
Insomnia	1	1 (3)	4	3 (11)
Lethargy	3	3 (9)	2	2 (7)
Rash	2	2 (6)	3	3 (11)
Acid reflux	4	4 (12)	0	0 (0)
Depressed mood	3	3 (9)	1	1 (4)
Anxiety	3	2 (6)	0	0 (0)
Bleeding	2	2 (6)	1	1 (4)
Frequent urination	1	1 (3)	2	2 (7)
Increased appetite	3	3 (9)	0	0 (0)
Increased thirst	3	3 (9)	0	0 (0)
Limb pain	0	0 (0)	3	3 (11)
Restlessness	1	1 (3)	2	2 (7)
Tinnitus	1	1 (3)	2	1 (4)

## Discussion

The main finding in this phase 2a trial was that in a group of manic and hypomanic patients, the addition of ebselen to ongoing anti-manic pharmacotherapy lowered scores on mania rating scales numerically more than the addition of placebo. In the case of the clinician-rated CGI-S scale, the difference favouring ebselen at the end of the final week of treatment was statistically significant. The same trend was apparent on the clinician-rated YMRS and the self-rated ASRM scales, although the decrease in symptomatic rating associated with ebselen addition was not statistically significantly different from placebo.

The mania rating scales also showed a pattern in which the placebo-treated participants demonstrated a steady improvement over the 3-week treatment period, which continued into follow-up at week 4. In contrast, the ebselen-treated participants showed higher scores in week 4 than in week 3. The latter finding is of interest because it suggests that withdrawal of ebselen in week 3 may have caused some loss of therapeutic benefit, supporting the conclusion that ebselen was active in the treatment of mania. The treatment of mania can sometimes result in increased depressive symptomatology. It is therefore worth noting that ebselen did not increase scores on the HDRS or the QIDS-SR.

The majority of the patients were taking antipsychotic drug treatment, which is the mainstay of the current pharmacotherapy of mania (Goodwin et al. 2016). However, it was not possible to control for dosing and changes in antipsychotic medication during the study which is an important limitation. Several participants were also taking anticonvulsant mood stabilisers, notably lamotrigine and valproate. While lamotrigine is not thought to be useful in the treatment of acute mania (being used principally to treat and prevent bipolar depression), valproate has been shown to be therapeutically effective for this indication (Cipriani et al. 2011; Goodwin et al. 2016). In addition, among its various pharmacological properties, valproate possesses the ability to inhibit inositol metabolism (see Rosenberg 2007); this mechanism of action could theoretically overlap with the lithium-like effects of ebselen. We therefore thought it of interest to exclude valproate-treated participants in a post hoc analysis. This revealed a somewhat stronger anti-manic treatment effect of ebselen, which was now of borderline statistical significance (*p* = 0.054), to lower symptom scores at 3 weeks on the YMRS, the primary outcome measure of the trial.

In addition, it is possible that the anti-manic effect of ebselen is not apparent when patients are taking another anti-manic mood stabiliser. However, this was a post hoc finding which needs prospective replication. With this caveat, our data suggest that while ebselen may potentiate the anti-manic effects of antipsychotic drugs, it may not produce additional therapeutic benefit when combined with valproate in this patient population.

Our study was double-blind, and placebo-controlled but otherwise pragmatic. The findings therefore need to be assessed within the important limitations of this kind of investigation. In particular, the great majority of patients were receiving concomitant pharmacological treatment for their current manic episode, which accounts for the reasonable therapeutic response seen overall. Connected to this, it was often not possible to enrol the more severely ill patients until they had started to show clinical improvement, due to the capacity needed to give full informed consent. Results of guesses by both researchers and participants of treatment allocation showed that the blinding of the study was well maintained (Supplementary Material, Table 3).

Our protocol called for an optional blood sample during the second week of ebselen treatment to determine ebselen plasma levels but in fact a significant number of participants declined this intervention; assays on the available samples have not currently been completed because of the impact of the COVID pandemic. However, it will be important in future studies to correlate ebselen blood levels with clinical response. Also testing for blood levels of concomitantly administered drugs will be necessary to exclude the possibility that ebselen, for example, might increase plasma levels of antipsychotic drugs.

The presence of active pharmacological treatment in addition to ebselen and placebo is likely to have made our proposed six-point difference on the YMRS unrealistic. In the subgroup of participants not receiving valproate, ebselen treatment produced an adjusted mean benefit over placebo of around three and a half points on the YMRS at week 3, so future trials might be powered to detect a difference of this order. Further studies to establish the potential benefits of ebselen in mania will also need to control auxiliary pharmacological treatments more tightly. However, our pragmatic design did make it feasible to recruit the 60 participants from routine NHS practice within a reasonable time frame. Moreover, participant feedback showed that the trial was well received and enjoyable with nearly all participants requesting a final report, allocation unblinding and the willingness to be contacted about future research.

Our studies confirm findings from previous clinical trials of ebselen both in healthy participants and in those with neurological disease, which indicate that the drug has a good safety profile with an incidence and severity of AEs very similar to placebo (Yamaguchi et al. 1998; Lynch and Kil 2009; Kil et al. 2017). This side effect profile clearly distinguishes ebselen from lithium and, in conjunction with the present findings, is a compelling reason for undertaking further trials with ebselen in patients with mood disorders. An appropriate next step might be a placebo-controlled trial of ebselen as a monotherapy in acutely manic patients (for example, see Keck et al. 2003).

## Electronic supplementary material

ESM 1 (DOCX 188 kb)

## References

[CR1] Agam G, Bersudsky Y, Berry GT, Moechars D, Lavi-Avnon Y, Belmaker RH (2009). Knockout mice in understanding the mechanism of action of lithium. Biochem Soc Trans.

[CR2] Altman EG, Hedeker D, Perterson JL, Davis JM (1997). The Altman Self-Rating Mania Scale. Biol Psychiatry.

[CR3] American Psychiatric Association. (2013). Diagnostic and statistical manual of mental disorders (5th ed.). 10.1176/appi.books.9780890425596

[CR4] Atack JR (1996). Inositol monophosphatase, the putative therapeutic target for lithium. Brain Res Rev.

[CR5] Berridge MJ, Downes CP, Hanley MR (1989). Neural and developmental actions of lithium: a unifying hypothesis. Cell.

[CR6] Chou JCY, Czobor P, Charles O, Tuma I, Winsberg B, Allen MH, Trujillo M, Volavka J (1999). Acute mania: haloperidol dose and augmentation with lithium or lorazepam. J Clin Psychopharmacol.

[CR7] Cipriani A, Barbui C, Salanti G, Rendell J, Brown R, Stockton S, Purgato M, Spineli LM, Goodwin GM, Geddes JR (2011). Comparative efficacy and acceptability of antimanic drugs in acute mania: a multiple-treatments meta-analysis. Lancet.

[CR8] Cousins DA, Squarcina L, Boumezbeur F, Young AH, Bellivier F (2020). Lithium: past, present, and future. Lancet Psychiatry.

[CR9] Geddes JR, Miklowitz DJ (2013). Treatment of bipolar disorder (2013). Lancet.

[CR10] Goodwin GM, Haddad PM, Ferrier IN, Aronson JK, Barnes TRH, Cipriani A, Coghill DR, Fazel S, Geddes JR, Grunze H, Holmes EA (2016). Evidence-based guidelines for treating bipolar disorder: revised third edition recommendations from the British Association for Psychopharmacology. J Psychopharmacol.

[CR11] Guy W (1976). Clinical global impressions. ECDEU Assessment Manual for Psychopharmacology—Revised.

[CR12] Haggarty SJ, Karmacharya R, Perlis RH (2020). Advances towards precision medicine for bipolar disorder: mechanisms and molecules. Mol Psychiatry (in press).

[CR13] Hamilton M (1960). A rating scale for depression. J Neurol Neurosurg Psychiatry.

[CR14] Harrison PJ, Cowen PJ, Burns T, Fazel M (2018). Shorter Oxford Textbook of Psychiatry.

[CR15] Keck PE, Marcus R, Tourkodimitris S, Ali M, Liebeskind A, Saha A, Ingenito G, Aripiprazole Study Group (2003). A placebo-controlled, double-blind study of the efficacy and safety of aripiprazole in patients with acute bipolar mania. Am J Psychiatr.

[CR16] Kil J, Pierce C, Tran H, Gu R, Lynch ED (2007). Ebselen treatment reduces noise induced hearing loss via the mimicry and induction of glutathione peroxidase. Hear Res.

[CR17] Kil J, Lobarinas E, Spankovich C, Griffiths SK, Antonelli PJ, Lynch ED, Le Prell CG (2017). Safety and efficacy of ebselen for the prevention of noise-induced hearing loss: a randomised, double-blind, placebo-controlled, phase 2 trial. Lancet.

[CR18] Klein E, Bental E, Lerer B, Belmaker RH (1984). Carbamazepine and haloperidol v placebo and haloperidol in excited psychoses: a controlled study. Arch Gen Psychiatry.

[CR19] Lukasiewicz M, Gerard S, Besnard A, Falissard B, Perrin E, Sapin H, Tohen M, Reed C, Azorin JM, Emblem Study Group (2013). Young Mania Rating Scale: how to interpret the numbers? Determination of a severity threshold and of the minimal clinically significant difference in the EMBLEM cohort. Int J Methods Psychiatr Res.

[CR20] Lynch E, Kil J (2009). Development of ebselen, a glutathione peroxidase mimic, for the prevention and treatment of noise-induced hearing loss. Semin Hear.

[CR21] Masaki C, Sharpley AL, Godlewska BR, Berrington A, Hashimoto T, Singh N, Vasudevan SR, Emir UE, Churchill GC, Cowen PJ (2016). Effects of the potential lithium-mimetic, ebselen, on brain neurochemistry: a magnetic resonance spectroscopy study at 7 tesla. Psychopharmacology.

[CR22] Masaki C, Sharpley AL, Cooper CM, Godlewska BR, Singh N, Vasudevan SR, Harmer CJ, Churchill GC, Sharp T, Rogers RD, Cowen PJ (2016). Effects of the potential lithium-mimetic, ebselen, on impulsivity and emotional processing. Psychopharmacology.

[CR23] Parrott AC, Hindmarsh I (1980). The Leeds Sleep Evaluation Questionnaire in psychopharmacological investigations-a review. Psychopharmacology.

[CR24] Rosenberg G (2007). The mechanisms of action of valproate in neuropsychiatric disorders: can we see the forest for the trees?. Cell Mol Life Sci.

[CR25] Rush AJ, Trivedi MH, Ibrahim HM, Carmody TJ, Arnow B, Klein DN, Markowitz JC, Ninan PT, Kornstein S, Manber R, Thase ME, Kocsis JH, Keller MB (2003). The 16-Item Quick Inventory of Depressive Symptomatology (QIDS), clinician rating (QIDS-C), and self-report (QIDS-SR): a psychometric evaluation in patients with chronic major depression. Biol Psychiatry.

[CR26] Singh N, Halliday AC, Thomas JM, Kuznetsova OV, Baldwin R, Woon EC, Aley PK, Antoniadou I, Sharp T, Vasudevan SR, Churchill GC (2013). A safe lithium mimetic for bipolar disorder. Nat Commun.

[CR27] Singh N, Sharpley AL, Emir UE, Masaki C, Herzallah MM, Gluck MA, Sharp T, Harmer CJ, Vasudevan SR, Cowen PJ, Churchill GC (2016). Effect of the putative lithium mimetic ebselen on brain myo-inositol, sleep, and emotional processing in humans. Neuropsychopharmacology.

[CR28] Yamaguchi T, Sano K, Takakura K, Saito I, Shinohara Y, Asano T, Yasuhara H (1998). Ebselen in acute ischemic stroke: a placebo-controlled, double-blind clinical trial. Stroke.

[CR29] Young RC, Biggs JT, Ziegler VE, Meyer DA (1978). A rating scale for mania: reliability, validity and sensitivity. Br J Psychiatry.

[CR30] Zivanovic O (2017). Lithium: a classic drug frequently discussed, but, sadly, seldom prescribed!. Aust N Z J Psychiatry.

