# Supplementary material for: A phase 2a randomised, double-blind, placebo-controlled, parallel-group, add-on clinical trial of ebselen (SPI-1005) as a novel treatment for mania or hypomania
Source: Psychopharmacology (Berl). 2020 Sep 9;237(12):3773–82. doi: 10.1007/s00213-020-05654-1 (PMC7683468; doi:10.1007/s00213-020-05654-1)
Supplement: Supplementary file 1 — (DOCX 188 kb) [file 213_2020_5654_MOESM1_ESM.docx]

Online Resource 1

Psychopharmacology

**Phase 2a investigation of ebselen (SPI-1005) in the treatment of mania and hypomania: A randomised, double-blind, placebo-controlled, parallel-group, add-on clinical trial**

Ann L Sharpley^1,2^ , Clare Williams^1,2^, Adele A Holder^1,2^, Beata R Godlewska^1,2^, Nisha Singh^1^, Milensu Shanyinde^3^, Orla Macdonald^2^ and Philip J Cowen^1,2^

1. Department of Psychiatry, University of Oxford, UK
2. Oxford Health NHS Foundation Trust, Warneford Hospital, Oxford, UK
3. Nuffield Department of Primary Care Health Sciences, University of Oxford, UK

*Corresponding Author*

Philip J Cowen, Neurosciences Building, Dept. of Psychiatry, University of Oxford, Warneford Hospital, Oxford, OX3 7JX, UK.

Email: [phil.cowen@psych.ox.ac.uk](mailto:ann.sharpley@psych.ox.ac.uk)

Phone: 01865 618311

Secondary Outcomes

Hamilton Depression Rating Scale- 17 item (HDRS 17)

High responses on clinician rated HDRS indicate high severity of depressive symptoms, thus a negative change from baseline indicates improvement in depressive symptoms.

HDRS change from baseline

Supplementary Figure 1: Non-adjusted mean (SEM) change from baseline scores on the Hamilton Depression Rating Scale (HDRS) following the addition of ebselen (600 mg bd) (n= 27) or placebo (n= 33) to the treatment of patients with mania/hypomania. There were no statistically significant differences at any time-points.

Quick Inventory of Depressive Symptomology-Self-Report (QIDS-SR-_16_)

High responses on the self-rated QIDS-SR_-16_ indicate presence of severe depressive symptoms, thus a negative change from baseline indicates improvement in depressive symptoms.

Supplementary Figure 2: Non-adjusted mean (SEM) change from baseline scores on the QIDS-SR_-16_ following the addition of ebselen (600 mg bd) (n= 27) or placebo (n= 33) to the treatment of patients with mania/hypomania. There were no statistically significant differences at any time-points.

Supplementary Table 1: Clinician-rated Clinical Global Impressions-Improvement (CGI-I) Scale

High responses on the clinician-rated CGI-I indicate worse symptoms of illness.

| Time-point | Ebselen  N | Mean (SD)  Range | Placebo  N | Mean (SD)  Range | Ebselen vs Placebo  1Adjusted difference in mean global improvement score [95%CI] *p* value |
| --- | --- | --- | --- | --- | --- |
| Week 1 | 25 | 3.0 (1.1)  1-6 | 32 | 3.2 (1.1)  2-6 | -0.04 [-0.68 to 0.60] 0.9030 |
| Week 2 | 24 | 3.2 (1.3)  1-6 | 29 | 3.3 (1.2)  2-5 | -0.11 [-0.76 to 0.55] 0.7538 |
| Week 3 | 22 | 2.7 (1.2)  1-5 | 29 | 3.1 (1.2)  1-6 | -0.28 [-0.96 to 0.39] 0.4109 |
| Week 4 | 20 | 3.1 (1.7)  1-6 | 28 | 3.5 (1.5)  2-7 | -0.31 [-1.01 to 0.39] 0.3818 |

There were no statistically significant differences between groups in terms of CGI-I at each time point.

**1**Adjusted for, severity of manic symptoms on YMRS (hypomania/mania), gender, psychosis, time and time*randomised group interaction. Random effect accounted for repeated measures on the same subject

### Leeds Sleep Evaluation Questionnaire (LSEQ)

The LSEQ contains 10 questions relating to four consecutive aspects of sleep; getting to sleep (GTS), quality of sleep (QOS), awake following sleep (AFS) and behaviour following wakening (BFW). Each question is assessed on a visual analogue scale (100‐mm‐line) with two extreme states 0 (difficulty) to 10 (not difficult). Each question is scored out of 10 with GTS and BFW having 3 questions and QOS and AFS having 2 questions. Thus, the minimum score for each domain will be 0 and maximum score ranging between 20 to 30.

Supplementary Table 2: Leeds Sleep Evaluation Questionnaire (LSEQ)

|  | **Ebselen** |  |  |  | **Placebo** |  |  |  |  | **95% CI** | |  |
| --- | --- | --- | --- | --- | --- | --- | --- | --- | --- | --- | --- | --- |
|  | **n** | **Mean** | **SD** | **Range** | **n** | **Mean** | **SD** | **Range** | **Adjusted**  **Mean diff** | **95% CI**  **LCL** | **95% CI**  **UCL** | ***p* value*** |
| **GTS** |  |  |  |  |  |  |  |  |  |  |  |  |
| **Week 1** | 26 | 17.6 | 7.0 | 5-30 | 32 | 17.5 | 5.5 | 6-30 | 0.27 | -2.77 | 3.31 | 0.8630 |
| **Week 2** | 23 | 17.7 | 6.0 | 8-29 | 29 | 18.7 | 6.2 | 7-30 | -1.29 | -4.46 | 1.89 | 0.4269 |
| **Week 3** | 21 | 20.1 | 6.5 | 8-30 | 28 | 19.2 | 6.7 | 6-30 | 0.86 | -2.41 | 4.13 | 0.6063 |
| **Week 4** | 19 | 18.2 | 4.8 | 9-27 | 27 | 18.4 | 5.0 | 10-30 | -0.42 | -3.78 | 2.93 | 0.8057 |
| **QOS** |  |  |  |  |  |  |  |  |  |  |  |  |
| **Week 1** | 26 | 9.6 | 4.3 | 0-19 | 32 | 12.2 | 4.7 | 2-20 | -2.57 | -4.69 | -0.46 | 0.0172 ***** |
| **Week 2** | 23 | 11.3 | 3.9 | 4-20 | 29 | 11.9 | 4.1 | 4-19 | -1.14 | -3.34 | 1.06 | 0.3109 |
| **Week 3** | 21 | 13.7 | 3.6 | 7-20 | 28 | 10.6 | 4.5 | 2-20 | 2.88 | 0.62 | 5.14 | 0.0126 ***** |
| **Week 4** | 18 | 11.9 | 2.6 | 8-18 | 27 | 11.2 | 4.2 | 0-20 | 0.38 | -1.96 | 2.73 | 0.7492 |
| **AFS** |  |  |  |  |  |  |  |  |  |  |  |  |
| **Week 1** | 26 | 10.1 | 5.2 | 0-20 | 32 | 11.3 | 4.7 | 2-20 | -0.94 | -3.14 | 1.26 | 0.4003 |
| **Week 2** | 23 | 11.6 | 4.6 | 5-20 | 29 | 10.4 | 4.2 | 3-20 | -1.25 | -1.05 | 3.55 | 0.2878 |
| **Week 3** | 21 | 11.2 | 4.4 | 2-20 | 28 | 10.9 | 4.2 | 4-20 | 0.30 | -2.07 | 2.66 | 0.8068 |
| **Week 4** | 18 | 10.8 | 3.0 | 5-17 | 27 | 10.1 | 4.4 | 2-20 | 0.53 | -1.94 | 2.99 | 0.6747 |
| **BFW** |  |  |  |  |  |  |  |  |  |  |  |  |
| **Week 1** | 26 | 15.4 | 7.8 | 2-29 | 32 | 18.4 | 6.5 | 7-29 | -2.64 | -5.71 | 0.43 | 0.0918 |
| **Week 2** | 23 | 18.3 | 5.6 | 5-30 | 29 | 15.5 | 5.7 | 5-30 | 2.24 | -0.97 | 5.44 | 0.1710 |
| **Week 3** | 21 | 17.2 | 6.2 | 2-29 | 28 | 16.3 | 5.6 | 6-30 | 0.50 | -2.80 | 3.79 | 0.7677 |
| **Week 4** | 18 | 17.4 | 6.4 | 6-30 | 27 | 14.4 | 5.0 | 4-25 | 2.48 | 0.94 | 5.90 | 0.1545 |

***** There were differences between groups in QOS at week 1 with ebselen showing a statistically significant reduction in QOS, *p*=0.0172 and at week 3, with ebselen showing a statistically significant improvement in QOS, *p*=0.0126. There were no statistically significant differences at other time points or in other domains (GTS, AFS and BFA).

Mixed effects linear model adjusting for baseline values.

Abbreviations: GTS=Getting to Sleep; QOS=Quality of Sleep; AFS=Awake following Sleep; BFW= Behaviour following wakening

Allocation Concealment

At the follow up visit (or earlier if discontinued) the researcher, participant and partner/relative/caregiver were asked to guess the allocation of treatment (ebselen or placebo). In addition, they are asked to gauge the confidence of their choice, measured on VAS scale (0 to 100). Higher values reflect greater certainty of guess.

Supplementary Table 3: Allocation Concealment

| Allocation |  | **Placebo**  **n = 33** | **Ebselen**  **n = 27** |
| --- | --- | --- | --- |
|  |  |  |  |
| **Researcher** | Placebo | 20 (61) | 13 (48) |
| Certainty of guess | Median [IQR] | 40 [25 to 48] | 32 [17 to 56] |
|  | Ebselen | 11 (33) | 12 (44) |
| Certainty of guess | Median [IQR] | 20 [10 to 50] | 40 [24 to 46] |
|  | Not reported | 2 (6) | 2 (7) |
|  |  |  |  |
| **Participant** | Placebo | 11 (33) | 9 (33) |
| Certainty of guess | Median [IQR] | 50 [50 to 90] | 50 [50 to 70] |
|  | Ebselen | 20 (61) | 17 (63) |
| Certainty of guess | Median [IQR] | 71 [45 to 90] | 60 [50 to 75] |
|  | Not reported | 2 (6) | 1 (4) |
|  |  |  |  |
| **Partner/relative/Caregiver** | Placebo | 5 (15) | 7 (26) |
| Certainty of guess | Median [IQR] | 69 [30 to 90] | 27 [0, 70] |
|  | Ebselen | 2 (6) | 1 (4) |
| Certainty of guess | Median [IQR] | 93 [-] | 51 [-] |
|  | Not reported | 26 (79) | 19 (70) |

(%)

[IQR]= Inter Quartile range

Participant Feedback Quotes

A small sample of feedback quotes:

- It was an unbelievably positive, pleasant experience. Dr. X has been hugely supportive - way and beyond the call of duty
- Involvement in the trial was a hassle-free experience, thank you for continuing to research treatment options for bipolar
- I appreciated the systematic yet human-centred approach taken by Y, as well as enthusiasm related to the research shown by others I met
- This research project was, is and will be first class, thoroughly enjoyed being a part of this study and Y’s company.
